# Biochemical and genetic characterization of a novel metallo-β-lactamase from marine bacterium *Erythrobacter litoralis* HTCC 2594

**DOI:** 10.1038/s41598-018-19279-0

**Published:** 2018-01-16

**Authors:** Xia-Wei Jiang, Hong Cheng, Ying-Yi Huo, Lin Xu, Yue-Hong Wu, Wen-Hong Liu, Fang-Fang Tao, Xin-Jie Cui, Bei-Wen Zheng

**Affiliations:** 1 College of Basic Medical Sciences, Zhejiang Chinese Medical University, Hangzhou, China; 2 Key Laboratory of Marine Ecosystem and Biogeochemistry, Second Institute of Oceanography, State Oceanic Administration, Hangzhou, China; 3 College of Life Sciences, Zhejiang Sci-Tech University, Hangzhou, China; 4 State Key Laboratory for Diagnosis and Treatment of Infectious Diseases, Collaborative Innovation Center for Diagnosis and Treatment of Infectious Diseases, The First Affiliated Hospital, School of Medicine, Zhejiang University, Hangzhou, China

## Abstract

Metallo-β-lactamases (MBLs) are a group of enzymes that can inactivate most commonly used β-lactam-based antibiotics. Among MBLs, New Delhi metallo-β-lactamase-1 (NDM-1) constitutes an urgent threat to public health as evidenced by its success in rapidly disseminating worldwide since its first discovery. Here we report the biochemical and genetic characteristics of a novel MBL, ElBla2, from the marine bacterium *Erythrobacter litoralis* HTCC 2594. This enzyme has a higher amino acid sequence similarity to NDM-1 (56%) than any previously reported MBL. Enzymatic assays and secondary structure alignment also confirmed the high similarity between these two enzymes. Whole genome comparison of four *Erythrobacter* species showed that genes located upstream and downstream of *elbla2* were highly conserved, which may indicate that *elbla*2 was lost during evolution. Furthermore, we predicted two prophages, 13 genomic islands and 25 open reading frames related to insertion sequences in the genome of *E. litoralis* HTCC 2594. However, unlike NDM-1, the chromosome encoded ElBla2 did not locate in or near these mobile genetic elements, indicating that it cannot transfer between strains. Finally, following our phylogenetic analysis, we suggest a reclassification of *E*. *litoralis* HTCC 2594 as a novel species: *Erythrobacter* sp. HTCC 2594.

## Introduction

Ever since the first antibiotic, penicillin, was used in the fight against infections caused by bacteria, there have been rising concerns regarding bacterial resistance to antibiotics acquired via the exposure of bacteria to sub-lethal quantities of antibiotics. As the number of different antibiotics used in clinical practice has increased, antibiotic resistance has become a serious problem worldwide. The emergence of genes encoding antibiotic-hydrolyzing enzymes, in particular, pose a major threat to human health. For example, β-lactamases enzymatically hydrolyze the β-lactam ring, the core structure of β-lactam-based antibiotics such as cephalosporins, cephamycins and carbapenems^1^. β-lactamases are divided into four different classes based on their nucleotide and amino acid sequences^2^. Due to their prevalence among clinical isolates, researches have mainly been focused on Class B β-lactamases (metallo-β-lactamases, MBLs)^3,4^. In addition, several MBL encoding genes have been found to be associated with mobile genetic elements (MGEs), enabling these genes to spread easily between species^5,6^. Another feature of MBLs is their dependence on metal ions (usually Zn^2+^) for catalytic activity. The requirement of metal ions as cofactors is a very common feature among enzymes^7^. To date, four subgroups (B1, B2, B3 and B4) of MBLs have been identified, based on their sequence homology^8^. Although MBLs of all the four subgroups need metal ions for their catalytic activity, they differ in the number of metal ions required. Generally, subgroups B1, B3 and B4 MBLs bind two Zn^2+^ in their active sites^9–14^, while subgroup B2 MBLs require only one Zn^2+^, the binding of an additional metal ion leads to inhibition of their activity^8,15^. However, BcII in subgroup B1 shows catalytic activity in the presence of both one and two Zn^2+^ ^16–18^.

The most common types of MBLs observed in clinical isolates are Imipenemase (IMP), Verona imipenemase (VIM) and New Delhi metallo-β-lactamase (NDM)^19^. IMP was initially discovered in 1994, in a clinical isolate of *Serratia marcescens* in which the enzyme conferred resistance to imipenem^20^. VIM was first identified in a carbapenem resistant isolate of *Pseudomonas aeruginosa* from an Italian patient in 1999^21^. NDM was first reported in 2009 in a carbapenem resistant *Klebsiella pneumoniae* strain isolated from a Swedish patient of Indian origin^22^. Since their first identification, all of the above mentioned enzymes have been found in various isolates worldwide. In addition, new types of MBLs are continuously emerging in both clinical isolates and environmental isolates^4,6,23,24^.

New Delhi metallo-β-lactamase-1 (NDM-1) shows very low similarities to other known β-lactamases, with the highest similarity (32.4%) being to VIM-1/VIM-2^22^. However, in our previous study, we performed a complete genome sequence analysis of a marine bacterium *Erythrobacter litoralis* HTCC 2594 (GenBank accession no. NC_007722) and reported an unexpectedly high similarity (56%) between NDM-1 and a β-lactamase II from *E. litoralis* HTCC 2594 (ElBla2)^25^. This report prompted Girlich *et al*. to characterize chromosomally encoded MBLs from five other *Erythrobacter* species (*E. citreus*, *E. flavus*, *E. longus*, *E. aquimaris* and *E. vulgaris*) in the hope to determine potential reservoirs of acquired MBLs^26^. However, they were unable to draw a connection between these species and plasmid-mediated carbapenemases spreading worldwide^26^. Interestingly, although ElBla2 was identified from *E. litoralis* HTCC 2594, it differed significantly from MBLs of other *Erythrobacter* species, sharing only 15% amino acid identity^26^. In addition, phylogenetic tree analysis showed that ElBla2 clustered in the same branch as clinical MBLs (NDM-1, VIM-1 and IMP-1) instead of with MBLs from its own genus members^25,26^. All these findings supported continued efforts into a comprehensive study on ElBla2.

In the present study, ElBla2 was first biochemically characterized to verify its ability to engender antibiotic resistance. We then aligned the amino acid sequences of ElBla2 with amino acid sequences of MBLs from different subgroups to identify the sites related to catalytic activity and metal ion binding. Whole genome comparison of four *Erythrobacter* species was performed and the gene arrangements around *elbla*2 were investigated in detail. By analyzing MGEs, we tried to estimate the gene transfer potential of *elbla*2. Finally, supported by the phylogenetic analysis, we propose a reclassification of the producer of ElBla2 as a novel species of genus *Erythrobacter*.

## Results

### Cloning and heterologous expression of ElBla2

Expression of ElBla2 in *E. coli* BL21 (DE3) conferred increased minimum inhibitory concentrations (MICs) to various β-lactams compared to the control strain (Table 1). The purification of the recombinant ElBla2 was visualized by SDS-PAGE (see Supplementary Fig. S1). The molecular weight of ElBla2 without its signal peptide was estimated to be 24.86 kDa (Expasy^27^), which was in accordance with the SDS-PAGE result.

**Table 1:** Antimicrobial susceptibility of *E. coli* BL21 harboring pET28a: *elbla*2 and *E. coli* BL21 harboring empty pET28a.

Antibiotics	BL21 (pET28a: *elbla*2)		BL21 (pET28a)	
	MIC (mg/L)	Interpretation	MIC (mg/L)	Interpretation
Ampicillin	≥32	R	≤2	S
Ampicillin/Sulbactam	≥32	R	≤2	S
Piperacillin/Tazobactam	≥128	R	≤4	S
Cefazolin	≥64	R	≤4	S
Cefotetan	≥64	R	≤4	S
Ceftazidime	≥64	R	≤1	S
Ceftriaxone	≥64	R	≤1	S
Cefepime	≤1	S	≤1	S
Aztreonam	≤1	S	≤1	S
Ertapenem	≥8	R	≤0.5	S
Imipenem	≥16	R	≤1	S

### Biochemical characterization

The enzymatic activity of the purified ElBla2 towards various antibiotic agents including amoxicillin, ampicillin, cefepime, meropenem, nitrocefin, cefotaxime and ceftazidime was determined by monitoring the hydrolysis of the antibiotic agents. ElBla2 reacted with amoxicillin, ampicillin, meropenem, cefotaxime and ceftazidime. Cefepime and nitrocefin, however, were not hydrolyzed by ElBla2. The comparison of kinetic parameters of ElBla2 and other MBLs (NDM-1^22^, CphA^28^ AIM-1^13^ and SPR-1^12^) for the hydrolysis of different types of β-lactams (amoxicillin, cefotaxime, meropenem and cefepime) are summarized in Table 2.

**Table 2:** Comparison of kinetic parameters of ElBla2 and MBLs from B1 (NDM-1), B2 (CphA), B3 (AIM-1) and B4 (SPR-1) subgroups.

Antibiotics	ElBla2			NDM-1^22^			CphA^28^			AIM-1^13^			SPR-1^12^		
	*K*_*m*_	*k*_cat_	*k*_cat_/*K*_*m*_	*K*_*m*_	*k*_cat_	*k*_cat_/*K*_*m*_	*K*_*m*_	*k*_cat_	*k*_cat_/*K*_*m*_	*K*_*m*_	*k*_cat_	*k*_cat_/*K*_*m*_	*K*_*m*_	*k*_cat_	*k*_cat_/*K*_*m*_
Amoxicillin	39 ± 9	1.4 ± 0.1	0.04 ± 0.01	NR^a^	NR	NR	NR	NR	NR	NR	NR	NR	NR	NR	NR
Cefotaxime	13 ± 4	0.9 ± 0.1	0.07 ± 0.02	10	6	0.58	34	0.1	0.0029	35 ± 4	170 ± 5	4.8	NR	NR	NR
Meropenem	19 ± 6	2.1 ± 0.2	0.11 ± 0.03	49	12	0.25	250	53	0.21	41 ± 4	760 ± 16	18	ND	ND	ND
Cefepime	ND^b^	ND	ND	77	13	0.17	NR	NR	NR	440 ± 60	37 ± 1	0.0084	NR	NR	NR

^a^NR: not reported.

^b^ND: not detected.

The *K*_*m*_ values are in (μM) and *k*_*cat*_ values are in (s^−1^).

### Sequence alignment and phylogenetic analysis

**Figure 1:**
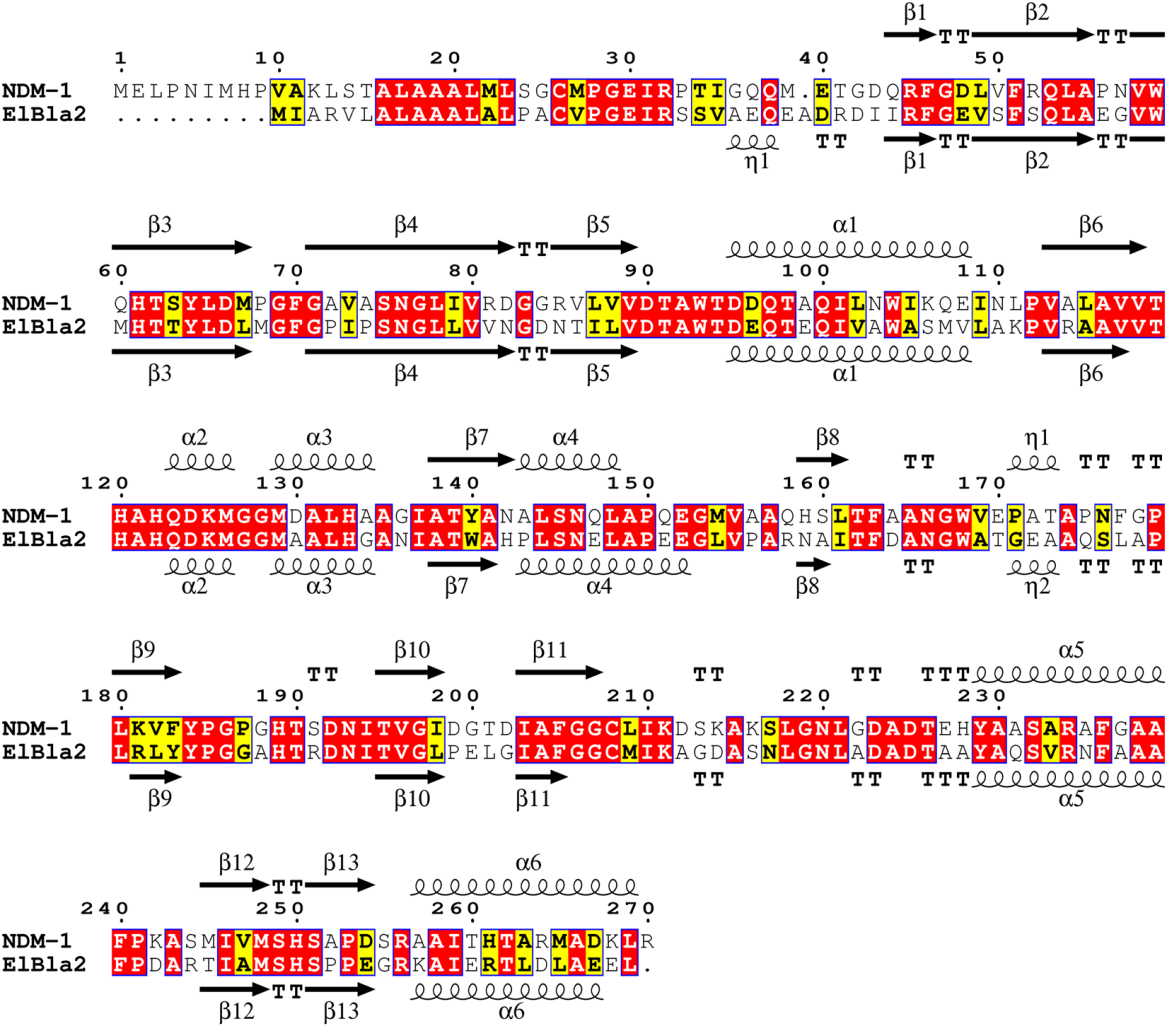
Aligned amino acid sequences and secondary structure of ElBla2 and NDM-1. Secondary structures are indicated with α (helices) and β (sheets). Positions that have a single, fully conserved residue are boxed in red. Conservation between groups of strongly similar properties are boxed in yellow.

**Figure 2:**
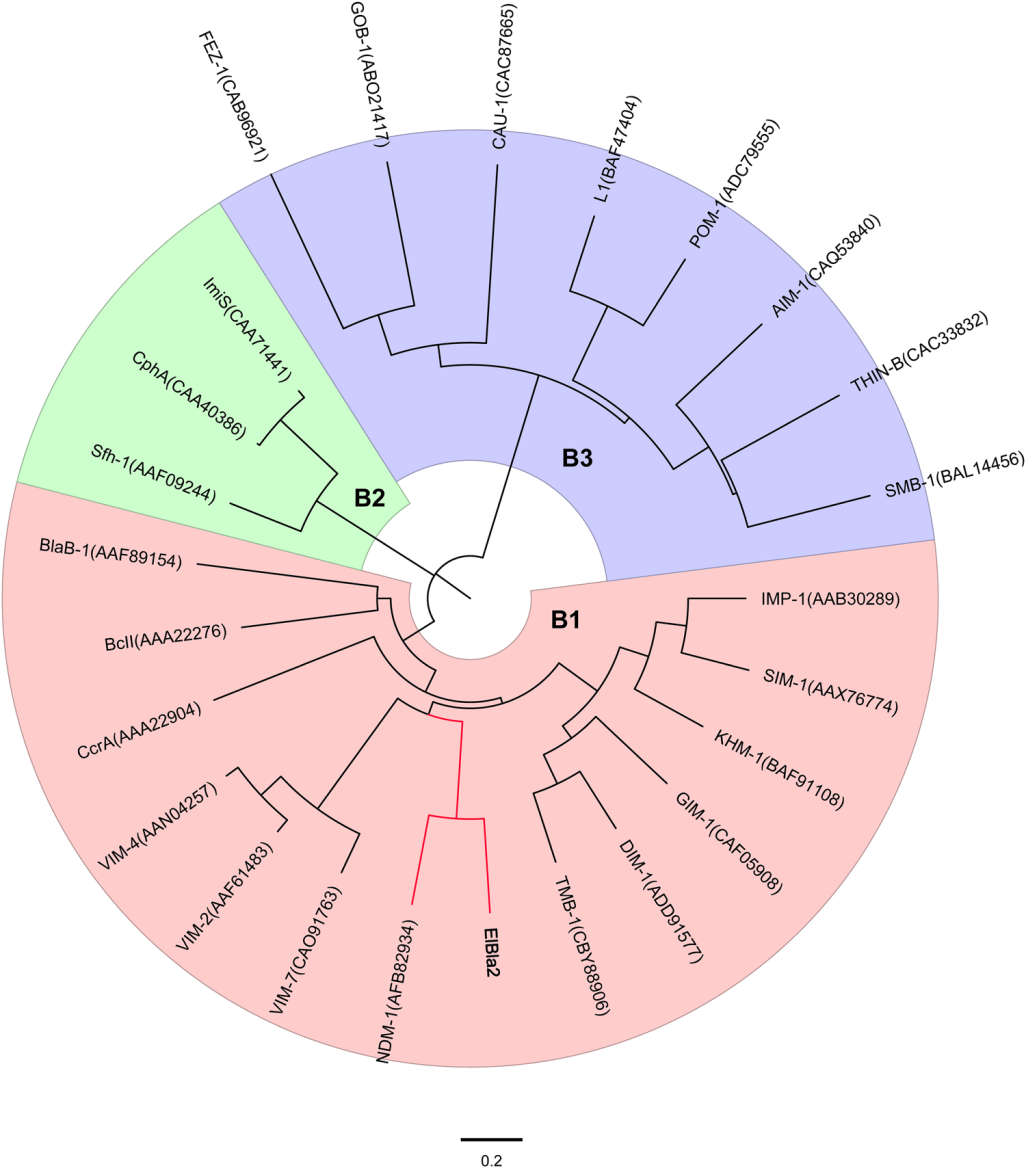
Evolutionary relationships of ElBla2 and other MBLs. The evolutionary history was inferred using the Neighbor-Joining method^54^. The optimal tree with a total branch length of 11.09452999 is shown. The tree is drawn to scale, with branch lengths in the same units as those of the evolutionary distances used to infer the phylogenetic tree. The evolutionary distances were computed using the Poisson correction method^66^ and represent the number of amino acid substitutions per site. The analysis involved 25 amino acid sequences of MBLs belonging to subgroup B1 (red), B2 (green) and B3 (purple). All ambiguous positions were removed for each sequence pair. There were a total of 336 positions in the final dataset. Evolutionary analyses were conducted in MEGA7^55^.

ElBla2 is encoded by a 786 bp ORF in the chromosome of *E. litoralis* HTCC 2594 and has a 56% amino acid sequence similarity to NDM-1. We used NDM-1 (Protein Data Bank code 3SPU) as a template to model the protein structure of ElBla2. The alignment and secondary structure of ElBla2 and NDM-1 are shown in Fig. 1. The alignment and comparison of ElBla2 and other MBLs are shown in Supplementary Table S1, Figs S2 and S3. In order to investigate the evolutionary relationship between ElBla2 and other MBLs, a phylogenetic tree was constructed using the amino acids sequences of various MBLs belonging to subgroups B1, B2 and B3 (Fig. 2).

### Comparative genomic study

**Figure 3:**
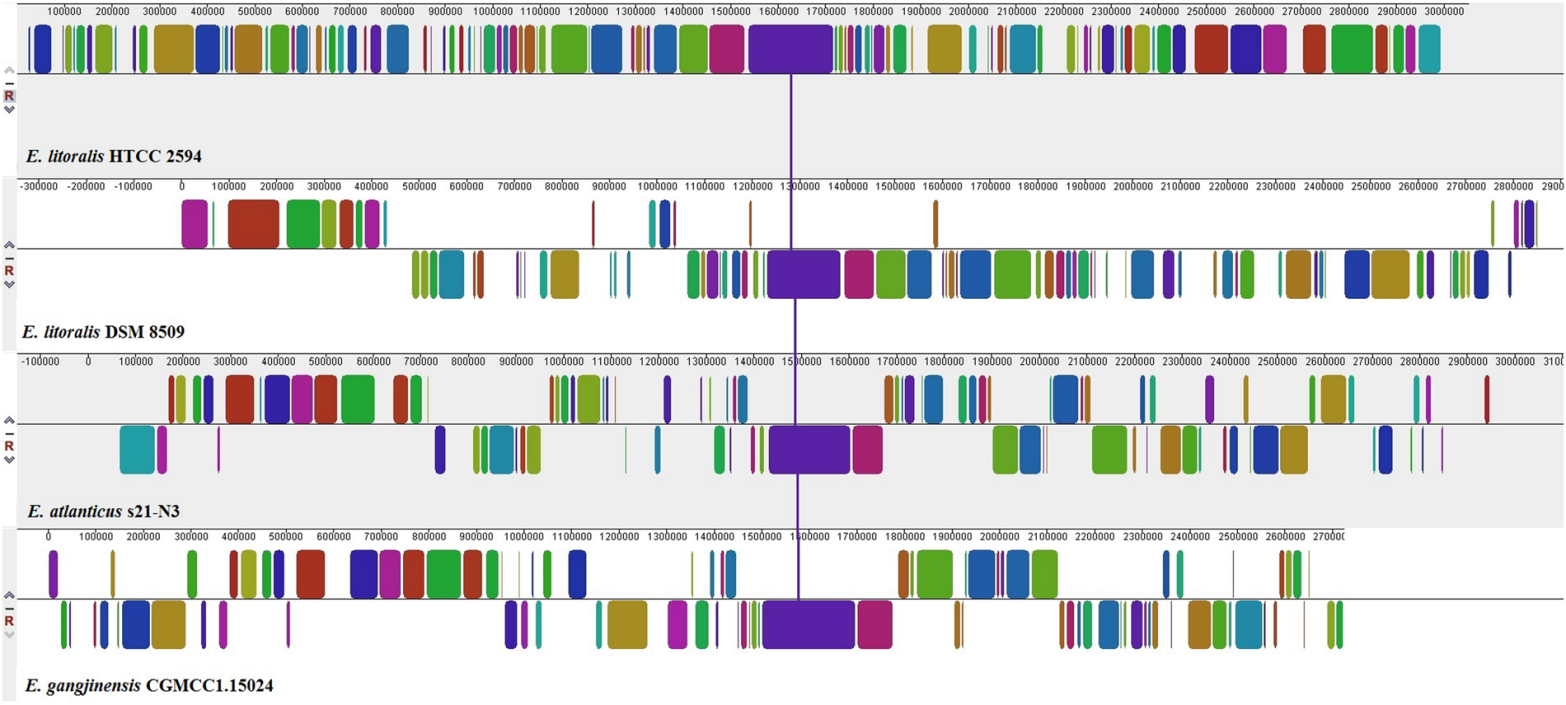
Progressive Mauve alignment of *E. litoralis* HTCC 2594, *E. litoralis* DSM 8509, *E. atlanticus* s21-N3 and *E. gangjinensis* CGMCC1.15024. Homologous alignments are represented as colored blocks. The gene encoding ElBla2 is located at position 1,569,105–1,569,800 bp in the genome of *E. litoralis* HTCC 2594.

**Figure 4:**
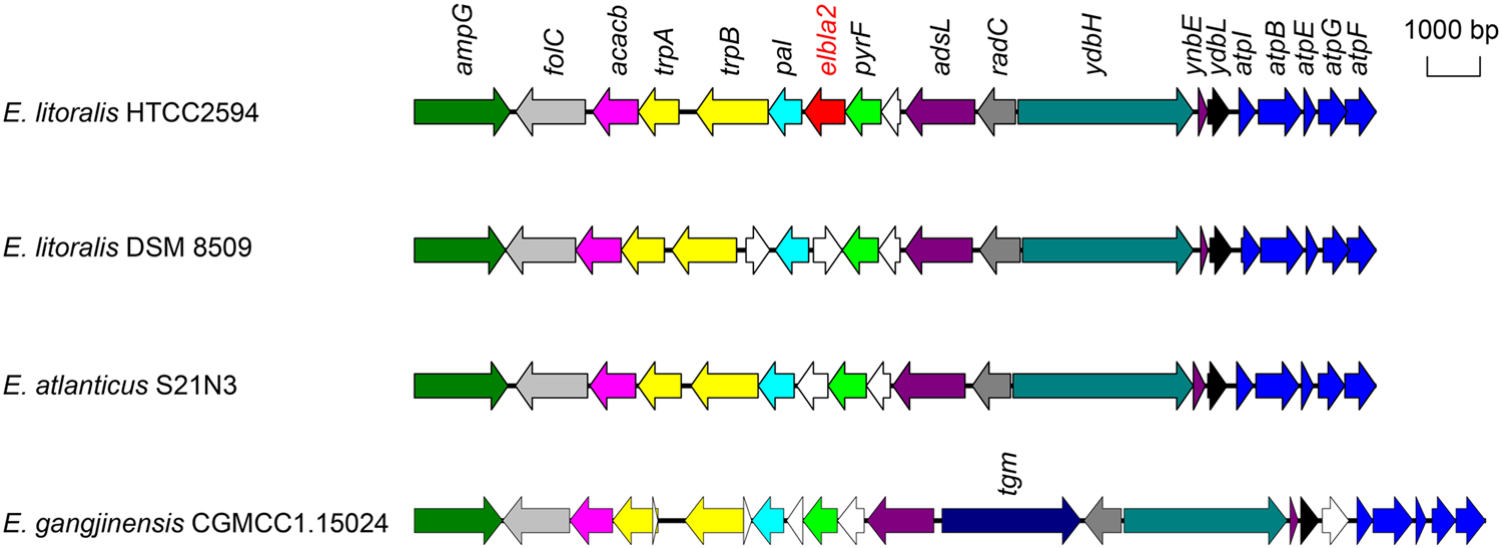
Structure of *elbla*2 locus in *E. litoralis* HTCC 2594 compared to *E. litoralis* DSM 8509, *E. atlanticus* s21-N3 and *E. gangjinensis* CGMCC1.15024 as inferred from RAST. Genes annotated with the same function were labeled in the same color, *elbla*2 is labeled in red and genes annotated as a hypothetical protein are labeled in white.

Thus far, there are four complete genomes of *Erythrobacter*, *E. atlanticus* s21-N3 (CP011310 and CP015441), *E. gangjinensis* CGMCC1.15024 (CP018097 and CP018098), *E. litoralis* DSM 8509 (CP017057) and *E. litoralis* HTCC 2594 (NC_007722) deposited in GenBank. All the four genomes were compared using ProgressiveMauve and significant homology was observed for the region around *elbla*2 (Fig. 3). Interestingly, the genes located upstream and downstream of *elbla*2 were highly similar in the four species (Fig. 4 and Supplementary Table S2).

### Mobile genetic elements

In an attempt to estimate the transfer potential of *elbla*2, the whole genome of *E. litoralis* HTCC 2594 was analyzed for the presence of MGEs including prophages, genomic islands (GIs), insertion sequences (ISs) and integrons. Two prophages and 13 GIs were identified in the genome (see Supplementary Figs S4, S5, Tables S3 and S4). A total of 25 ORFs related to ISs of different families were also found (Supplementary Table S5), but no integrons were detected.

## Discussion

NDM-1 has been the cause of great concern since it was first reported in 2009^22^. Given that the enzyme only shares a few similarities with other MBLs, researchers have endeavored to find its close relatives. In our previous study, we reported an unexpectedly high similarity in amino acid sequences between NDM-1 and ElBla2^25^. In the present work, a comprehensive analysis of both biochemical and genetic aspects of ElBla2 was performed. To our knowledge, no other MBL has been reported with such a high similarity to NDM-1.

ElBla2 was successfully expressed in *E. coli* BL21 (DE3) and antimicrobial susceptibility testing showed increased MICs to various β-lactams compared with the control strain (Table 1). High-level resistance to penicillins was detected in the ElBla2-producing strain, the highest MICs (≥128 mg L^−1^) were for piperacillin. The MICs of cephalosporins were also increased with the production of ElBla2, with the exception of cefepime. Furthermore, production of ElBla2 conferred resistance to carbapenems. MICs of ertapenem and imipemem were ≥8 mg L^−1^ and ≥16 mg L^−1^, respectively. However, the production of ElBla2 had no effect on the MIC of aztreonam. In addition, the purified ElBla2 showed enzymatic activity towards amoxicillin, ampicillin, meropenem, cefotaxime and ceftazidime, but not cefepime or nitrocefin. The inability of ElBla2 to hydrolyze nitrocefin is noteworthy since most of MBLs catalyze nitrocefin, as reported in other studies. However, there are exceptions. For example, Ferreira *et al*. revealed that two isolates of *Staphylococcus aureus*, one isolate of *S. epidermidis* and one isolate of *S. lugdunensis* which carried *blaZ* genes tested negative in nitrocefin tests^29^. Pitkala *et al*. found that 15 out of 19 *Staphylococcus* spp. isolates harboring *blaZ* genes showed a very weak reaction to nitrocefin^30^. Although the nitrocefin tests are commonly used tests for MBL detection, there are a large number of false-negative results according to different studies, indicating that not all MBLs can hydrolyze nitrocefin. ElBla2 showed lower *K*_*m*_ values for cefotaxime than for the other two substrates, indicating that ElBla2 binds tightly to cefotaxime (Table 2). In comparison, NDM-1, closely related to ElBla2 in terms of amino acid sequence, also exhibits low *K*_*m*_ values to most cephalosporins, especially to cefotaxime (*K*_*m*_ = 10 μM)^22^. However, the turnover rate (*k*_cat_) of ElBla2 for the different substrates was lower compared to that of NDM-1. The highest turnover rate of ElBla2 was observed for meropenem (2.1 ± 0.2 s^−1^) whereas the turnover rate by NDM-1 for meropenem is 5.6-fold higher^10^. The highest catalytic efficiency (*k*_cat_/*K*_*m*_) of ElBla2 (0.11 ± 0.03 s^−1^ μM^−1^) was also observed for meropenem (3.1-fold of amoxicillin and 1.6-fold of cefotaxime). This value was, however, also lower than the corresponding value of NDM-1.

The secondary protein structure of ElBla2 and NDM-1 are very similar (Fig. 1); both contain 13 β-sheets and six α-helices. Many active sites in NDM-1 are also found to be conserved in ElBla2, such as N220 which is involved in the interaction with the lactam carbonyl group and K211 which orients the negatively charged carboxylate common to β-lactam substrates^31^. Residues L65, M67 and W93 in NDM-1, which form a hydrophobic face that tightly interacts with the R1 phenyl group of the substrate, are not all conserved in ElBla2 since M67 is substituted by a leucine in ElBla2^31^. The alignment of ElBla2 to MBLs representing four subgroups of Class B β-lactamases indicated that ElBla2 harbors all the six conserved residues which forms the amino acid ligands in the two metal ion (Zn1 and Zn2) binding sites (see Supplementary Fig. S2). Recently, a number of MBLs from non-pathogenic bacteria and environments that have not been impacted by human activities were reported, such as LAR-1-LAR13 from the metagenome of remote Alaskan soil^32^, MIM-1 from *Novosphingobium pentaromativorans* and MIM-2 from *Simiduia agarivorans*^33^. Characterizations of these enzymes showed that they were efficient MBLs. For example, LAR-8 was found to be active towards penicillins and carbapenems^34^, while LAR-12 displayed strong carbapenemase activity^35^. Some of these enzymes are also bifunctional. For example, the enzymes MIM-1 and MIM-2 exhibit lactonase activity besides lactamase activity^36^. Comparison of amino acid sequences between ElBla2 and these environmentally-derived MBLs showed that ElBla2 shares relatively lower identity with these MBLs than with clinically-derived NDM-1 (see Supplementary Table S1). Furthermore, in order to predict whether ElBla2 has lactonase activity like MIM-1 and MIM-2, amino acid sequences of these three proteins were aligned. The result showed that two of the three conserved residues were absent in ElBla2 (see Supplementary Fig. S3). These residues are known to be critical for lactonase activity and are conserved in MIM-1, MIM-2 and other N-acyl homoserine lactonases (AHLases)^36^. The absence of these residues indicates that ElBla2 likely has no lactonase activity.

It has previously been reported that NDM-1 only shares low identity with other MBLs and forms a unique branch within subgroup B1^22^. However, with the discovery of ElBla2 in the marine bacterium *E. litoralis* HTCC 2594, the branch in subgroup B1 is populated by both NDM-1 and ElBla2 (Fig. 2). The similarity in amino acid sequences between ElBla2 and NDM-1 was first discovered by Zheng *et al*.^25^ and this finding instigated the study of chromosomally encoded MBLs from the genus *Erythrobacter*^26^. However, these endeavors failed to identify any MBLs from *Erythrobacter* spp. sharing high similarity with ElBla2, NDM-1 or any other MBL of clinical importance^26^; making ElBla2 unique. In recent years, the boom in whole genome sequencing projects has made the data pool of bacterial genomes more extensive. Even as an increasing number of species are being sequenced, no MBL with higher sequence similarity to NDM-1 than ElBla2 has yet been found. Following whole genome comparison analysis of four species of *Erythrobacter*, we observed that the gene arrangements around *elbla*2 were highly conserved in all of the *Erythrobacter* species included in the study (Fig. 4), suggesting that *elbla*2 may have been lost during evolution.

Many genes, especially those contributing to virulence and antibiotic resistance in bacteria, can be transmitted via various types of MGEs^37^. As a result, bacteria are able to acquire virulence and antibiotic resistance via horizontal gene transfer (HGT). Although numerous MGEs were identified in the genome of *E. litoralis* HTCC 2594 (see Supplementary Figs S4, S5 and Tables S3–S5), *elbla*2 was not located in or near any of these MGEs, indicating that it may unable to transfer among strains. Contrastingly, *bla*_NDM-1_ has always been reported to be located on a plasmid or embedded in the chromosome along with MGEs and can thus spread widely among different strains, making it an important public health issue^22,38^.

Generally, bacteria of the same species share high similarity in genome sequences. However, *elbla*2 was only located in the chromosome of *E. litoralis* HTCC 2594. In fact, another strain of *E. litoralis*, DSM 8509, does not encode any MBL that shows sequence similarity to *elbla*2. Therefore, a blast-analysis was performed between these two strains and surprisingly, many differences between their genomes were observed (see Supplementary Fig. S6). For this reason, the 16S rRNA gene sequences of HTCC 2594 and DSM 8509 were subsequently compared using BLASTN and the result showed a similarity of 98%. Though the original standard for 16 S rRNA gene sequence at which prokaryotic species delineate is a similarity of 97%^39^, a more relaxed cut-off value of 98.7–99% has recently been proposed^40^. The low similarity (98%) of 16 S rRNA gene sequence between HTCC 2594 and DSM 8509 may suggest that they belong to different species. To further test this assumption, we calculated the average nucleotide identity (ANI) values between them. ANI has been widely used to compare two prokaryotic genome sequences when classifying and identifying bacteria and ANI values of about 95–96% are considered to be the species boundary^41–43^. In accordance with the 16 S rRNA gene sequence similarity, the ANI value between HTCC 2594 and DSM 8509 (74.23%) is much lower than the species boundary. Supported by these results, we can conclude that HTCC 2594 and DSM 8509 are not of the same species. DSM 8509 is the type strain of *E. litoralis* species and the original publication reporting its isolation and identification emphasized its distinct phylogenetic position and phenotypic characteristics^44^. The publications related to HTCC 2594 on the other hand, only report the sequence of its whole genome and a characterization of its functional genes^45,46^. For these reasons, we propose to reclassify *E. litoralis* HTCC 2594 as a novel species: *Erythrobacter* sp. HTCC 2594.

In conclusion, we have successfully characterized a novel MBL, named ElBla2, from a marine bacterium. This novel enzyme is the MBL with the highest degree of amino acid sequence similarity to NDM-1 discovered thus far. Subsequent to the biochemical characterization of ElBla2, its secondary structure was aligned with that of NDM-1 to compare the active sites. The alignment of ElBla2 to MBLs representing the four subgroups of the Class B family indicated that ElBla2 harbors all the conserved residues related to the binding of two metal ions. Furthermore, gene arrangements of four species of *Erythrobacter* were compared and the results showed that the genes located upstream and downstream of *elbla*2 were highly conserved, indicating that *elbla*2 may have been lost during evolution. Also, *elbla*2 did not locate in or near MGEs, suggesting that it is unable to be transferred among strains. Finally, supported by the phylogenetic analysis we propose a reclassification of the producer of ElBla2, *E. litoralis* HTCC 2594, as a novel species: *Erythrobacter* sp. HTCC 2594.

## Methods

### Cloning of *elbla*2

DNA was synthesized according to the nucleic acid sequence of the gene encoding β-lactamase II in *E. litoralis* HTCC 2594 (*elbla*2) (GenBank accession NC_007722). The full-length of *elbla*2 was amplified from the synthesized DNA by PCR using the primers EL-F1 and EL-R (Table 3). However, expression of the full-length ElBla2 did not yield any detectable protein. Therefore, we predicted the presence of signal peptide cleavage sites in ElBla2 using SignalP 4.1 Server^47^ and found a potential cleavage site between position 28 and 29. According to the position of the cleavage site in ElBla2, the mature-length *elbla*2 was amplified using primers EL-F2 and EL-R (Table 3). Cloning of the mature-length *elbla*2 into a pET28a vector (Invitrogen) was performed as previously described^48^. The recombinant plasmid pET28a-*elbla*2 was verified by DNA sequencing for the presence of the *elbla*2 gene. Recombinant plasmid pET28a-*elbla*2 was transformed into *E. coli* BL21 (DE3) cells for production of ElBla2.

**Table 3:** PCR primers used in this study.

Primer name	Sequence(5′–3′)	Restriction site
EL-F1	TATACATATGGTGATCGCGCGGG	*Nde*I
EL-F2	ATATCATATGGAGCAGGAGGCCGAC	*Nde*I
EL-R	TATGCTCGAGCTAGAGTTCTTCGGC	*Xho*I

### Antimicrobial susceptibility testing

Antimicrobial susceptibility testing of *E. coli* BL21 harboring pET28a-*elbla*2 was determined using the VITEK 2 system employing panel AST-GN-13 (bioMerieux, France). The results were interpreted using the standards of the Clinical and Laboratory Standards Institute. *E. coli* BL21 harboring pET28a were used as controls.

### Enzymatic assay

ElBla2 was purified using the Ni-NTA affinity chromatography column (Qiagen, Germany) according to the manufacturer’s protocol. The purified protein was verified by 12% sodium dodecyl sulfate-polyacrylamide gel electrophoresis (SDS-PAGE) and the concentration of the protein was estimated using Bradford reagent^49^. The enzymatic activity of ElBla2 towards various β-lactam antibiotic agents was determined at 35 °C in Tris-HCl buffer (pH 7.4) using a Coulter DU 800 spectrophotometer (BecKman) as described elsewhere^48^. The details of reaction conditions are shown in the Supplementary Methods. The enzyme kinetics analysis for ElBla2 was performed by measuring enzyme velocity towards different concentrations of the substrates. The substrates and the concentrations used were amoxicillin (ranging from 20 μM to 500 μM), cefotaxime (ranging from 10 μM to 100 μM) and meropenem (ranging from 10 μM to 200 μM). Nonlinear regression curves for each substrate were drawn using the substrate concentrations and the enzyme velocity. Then the Lineweaver-Burk plot, which plots the reciprocal of substrate concentration vs. the reciprocal of enzyme velocity, was created for each substrate. The concentration of ElBla2 in the reaction was measured by the method of Bradford^49^ using bovine serum albumin as standard. The *K*_*m*_ and *k*_*cat*_ values were calculated according the Michaelis–Menten equation using GraphPad Software (GraphPad Inc., USA)^50^. All values were recorded in triplicate and the blank control was performed using the deactivated enzyme.

### Bioinformatics analysis

Protein structure modelling was conducted using the SWISS-MODEL Workspace^51^. Multiple sequence alignment was performed using Clustal Omega^52^ and the secondary structure information from the aligned sequence was rendered using ESPript 3.0^53^. The phylogenetic tree was constructed according to the Neighbor-Joining Method^54^ using Molecular Evolutionary Genetics Analysis (MEGA) 7.0 software^55^. BLASTN was used for the alignment of the whole genomes and the genome alignment was visualized by BLAST Ring Image Generator^56^. The genomic ANI was calculated using EzBioCloud^57^ with OrthoANIu algorithm^58^. Genomic comparison was conducted using ProgressiveMauve^59^. Genomes were annotated by Rapid Annotations using Subsystems Technology (RAST) server^60^. Prophage regions were predicted with the PHAge Search Tool Enhanced Release server^61,62^ and GIs were predicted by IslandViewer 4 using four different methods (IslandPick, IslandPath-DIMOB, SIGI-HMM, and Islander)^63^. Insertion sequences and integrons were predicted using ISsaga^64^ and IntegronFinder^65^, respectively.

## Electronic supplementary material

Supplementary Information
