# Supplementary material for: Biochemical and genetic characterization of a novel metallo-β-lactamase from marine bacterium Erythrobacter litoralis HTCC 2594
Source: Sci Rep. 2018 Jan 16;8:803. doi: 10.1038/s41598-018-19279-0 (PMC5770381; doi:10.1038/s41598-018-19279-0)
Supplement: Supplementary file 1 — Supplementary Information [file 41598_2018_19279_MOESM1_ESM.pdf]

Supplementary Information

Biochemical and genetic characterization of a novel metallo-β-lactamase from marine bacterium *Erythrobacter litoralis* HTCC 2594

Running title: Novel metallo-β-lactamase from *Erythrobacter litoralis* HTCC 2594

Xia-Wei Jiang[1#], Hong Cheng[2#], Ying-Yi Huo[2], Lin Xu[3], Yue-Hong Wu[2], Wen-Hong Liu[1], Fang-Fang Tao[1], Xin-Jie Cui[1], Bei-Wen Zheng[4*]

[1]College of Basic Medical Sciences, Zhejiang Chinese Medical University, Hangzhou, China

[2]Key Laboratory of Marine Ecosystem and Biogeochemistry, Second Institute of Oceanography, State Oceanic Administration, Hangzhou, China

[3]College of Life Sciences, Zhejiang Sci-Tech University, Hangzhou, China

[4]State Key Laboratory for Diagnosis and Treatment of Infectious Diseases, Collaborative Innovation Center for Diagnosis and Treatment of Infectious Diseases, The First Affiliated Hospital, School of Medicine, Zhejiang University, Hangzhou, China

*Correspondence and requests for materials should be addressed to B.Z. (email:zhengbw@zju.edu.cn)

[#]These two authors contribute equally to this work.

Methods

Reaction conditions of ElBla2 towards different substrates. The standard assay was performed as follows: 980 μl Tris-HCl buffer (pH 7.4), 100 μM substrates, and the enzyme in a final volume of 1 ml. The activity of the enzyme was determined at 35 °C by measuring it under the specific wavelength for each substrates using a Coulter DU 800 nucleic acid/protein analyzer (Beckman). The substrates and wavelengths used in this study were: amoxicillin (240 nm), ampicillin (235 nm), cefepime (264 nm), meropenem (298 nm), nitrocefin (500 nm), cefotaxime (265 nm) and ceftazidime (260 nm). The extinction coefficient for agent changes with temperatures and the types of solvents, so we didn't use the extinction coefficient for each substrate according to the references. Instead, we draw a standard curve for each substrate by preparing a serials of different concentrations of the substrate with the reaction buffer (Tris-HCl buffer, pH 7.4) and measuring their absorbance under its specific wavelength at 35 °C. Then we use this standard curve for each substrate to calculate the amount of the substrates in the enzymatic assay in our study. All values were determined in triplicate and the blank was performed using the deactivated enzyme.

Table S1. Pairwise sequence comparisons between ElBla2 and MBLs from non-pathogenic organisms.

	ElBla2	MIM-1	MIM-2	LRA-8	LRA-12
ElBla2	100%	28%	41%	27%	35%
MIM-1		100%	47%	41%	31%
MIM-2			100%	42%	33%
LRA-8				100%	30%
LRA-12					100%

Table S2. Gene arrangements around *elbla*2 in *E. litoralis* HTCC 2594, *E. litoralis* DSM 8509, *E. atlanticus* s21-N3 and *E. gangjinensis* CGMCC1.15024.

Species	Start	End	Annotation
E. litoralis HTCC 2594	1559710	1559147	ATP synthase F0 sector subunit b (EC 3.6.3.14)
	1560197	1559703	ATP synthase F0 sector subunit b' (EC 3.6.3.14)
	1560503	1560276	ATP synthase F0 sector subunit c (EC 3.6.3.14)
	1561336	1560548	ATP synthase F0 sector subunit a (EC 3.6.3.14)
	1561709	1561374	ATP synthase protein I
	1562267	1561878	Putative uncharacterized protein ydbL, may be related to amine metabolism
	1562459	1562286	Uncharacterized protein ynbE; probable lipoprotein STY1424
	1565746	1562525	Putative uncharacterized protein ydbH
	1565832	1566542	DNA repair protein RadC
	1566603	1567913	Adenylosuccinate lyase (EC 4.3.2.2)
	1567985	1568347	FIG00635921: hypothetical protein
	1568344	1569018	Orotidine 5'-phosphate decarboxylase (EC 4.1.1.23)
	1569015	1569800	Beta-lactamase
	1569814	1570443	Phosphoribosylanthranilate isomerase (EC 5.3.1.24)
	1570466	1571767	Tryptophan synthase beta chain (EC 4.2.1.20)
	1572093	1572872	Tryptophan synthase alpha chain (EC 4.2.1.20)
	1572883	1573731	Acetyl-coenzyme A carboxyl transferase beta chain (EC 6.4.1.2)
	1573829	1575118	Dihydrofolate synthase (EC 6.3.2.12) @ Folylpolyglutamate synthase (EC 6.3.2.17)
	1577016	1575241	AmpG permease
E. litoralis DSM 8509	1347610	1349325	AmpG permease
	1350633	1349326	Dihydrofolate synthase (EC 6.3.2.12) @ Folylpolyglutamate synthase (EC 6.3.2.17)
	1351494	1350643	Acetyl-coenzyme A carboxyl transferase beta chain (EC 6.4.1.2)
	1352303	1351491	Tryptophan synthase alpha chain (EC 4.2.1.20)

	1353683	1352466	Tryptophan synthase beta chain (EC 4.2.1.20)
	1353877	1354335	hypothetical protein
	1355042	1354398	Phosphoribosylanthranilate isomerase (EC 5.3.1.24)
	1355149	1355652	hypothetical protein
	1356355	1355675	Orotidine 5'-phosphate decarboxylase (EC 4.1.1.23)
	1356762	1356352	FIG00635921: hypothetical protein
	1358139	1356856	Adenylosuccinate lyase (EC 4.3.2.2)
	1359034	1358237	DNA repair protein RadC
	1359060	1362296	Putative uncharacterized protein ydbH
	1362407	1362562	Uncharacterized protein ynbE; probable lipoprotein STY1424
	1362595	1362987	Putative uncharacterized protein ydbL, may be related to amine metabolism
	1363182	1363550	ATP synthase protein I
	1363572	1364360	ATP synthase F0 sector subunit a
	1364412	1364639	ATP synthase F0 sector subunit c (EC 3.6.3.14)
	1364713	1365207	ATP synthase B' chain (EC 3.6.3.14)
	1365200	1365727	ATP synthase F0 sector subunit b (EC 3.6.3.14)
E. atlanticus s21-N3	1573742	1575478	AmpG permease
	1576915	1575578	Dihydrofolate synthase (EC 6.3.2.12) @ Folylpolyglutamate synthase (EC 6.3.2.17)
	1577806	1576958	Acetyl-coenzyme A carboxyl transferase beta chain (EC 6.4.1.2)
	1578611	1577832	Tryptophan synthase alpha chain (EC 4.2.1.20)
	1580034	1578817	Tryptophan synthase beta chain (EC 4.2.1.20)
	1580681	1580031	Phosphoribosylanthranilate isomerase (EC 5.3.1.24)
	1581298	1580753	hypothetical protein
	1582041	1581331	Orotidine 5'-phosphate decarboxylase (EC 4.1.1.23)
	1582457	1582038	hypothetical protein
	1583842	1582529	Adenylosuccinate lyase (EC 4.3.2.2)

	1584671	1583952	DNA repair protein RadC
	1584718	1588005	Putative uncharacterized protein ydbH
	1588002	1588244	Uncharacterized protein ynbE; probable lipoprotein STY1424
	1588266	1588652	Putative uncharacterized protein ydbL, may be related to amine metabolism
	1588795	1589124	ATP synthase protein I
	1589151	1589942	ATP synthase F0 sector subunit a
	1589989	1590213	ATP synthase F0 sector subunit c (EC 3.6.3.14)
	1590314	1590790	ATP synthase F0 sector subunit b' (EC 3.6.3.14)
	1590783	1591364	ATP synthase F0 sector subunit b
E. gangjinensis CGMCC1.15024	1654838	1656589	AmpG permease
	1657920	1656586	Dihydrofolate synthase (EC 6.3.2.12) @ Folylpolyglutamate synthase (EC 6.3.2.17)
	1658789	1657938	Acetyl-coenzyme A carboxyl transferase beta chain (EC 6.4.1.2)
	1659668	1658799	Tryptophan synthase alpha chain (EC 4.2.1.20)
	1659579	1659695	hypothetical protein
	1661416	1660232	Tryptophan synthase beta chain (EC 4.2.1.20)
	1661396	1661545	hypothetical protein
	1662204	1661554	Phosphoribosylanthranilate isomerase (EC 5.3.1.24)
	1662592	1662281	hypothetical protein
	1663270	1662596	Orotidine 5'-phosphate decarboxylase (EC 4.1.1.23)
	1663788	1663267	hypothetical protein
	1665188	1663875	Adenylosuccinate lyase (EC 4.3.2.2)
	1665336	1668140	Transglutaminase-like enzyme
	1668902	1668156	DNA repair protein RadC
	1668969	1672226	Putative uncharacterized protein ydbH
	1672274	1672468	Uncharacterized protein ynbE; probable lipoprotein STY1424
	1672497	1672877	Putative uncharacterized protein ydbL, may be related to amine metabolism

	1672949	1673455	hypothetical protein
	1673609	1673926	ATP synthase protein I
	1673962	1674759	ATP synthase F0 sector subunit a
	1674805	1675032	ATP synthase F0 sector subunit c (EC 3.6.3.14)
	1675135	1675608	ATP synthase F0 sector subunit b' (EC 3.6.3.14)
	1675601	1676185	ATP synthase F0 sector subunit b

Table S3. Prophage regions identified in *E. litoralis* HTCC 2594

Region	Length (kb)	Completeness	Total Proteins	Position	Possible phage	GC (%)
1	28.6	incomplete	21	157555-186251	PHAGE_Aggreg_S1249_NC_013597	59.32
2	13.8	questionable	18	2812951-2826784	PHAGE_Salmon_ST64B_NC_004313	67.59

Table S4. Genomic islands in *E. litoralis* HTCC 2594.

Genomic Island	Prediction Method	Island start	Island end	Length	Gene start	Gene end	Product	External Annotations
Gl-1	Islander	33863	38537	4674	34020	35177	hypothetical protein	
					35602	35787	hypothetical protein	
					36813	37406	hypothetical protein	
					37385	38254	DUF4747 domain-containing protein	
GI-2	Islander	157414	241464	84050	157555	158736	integrase	
					158949	159533	lytic transglycosylase	
					160143	160592	hypothetical protein	
					160669	164916	methylase	
					165021	167012	chromosome partitioning protein ParB	
					167179	168108	hypothetical protein	
					168120	168548	hypothetical protein	
					168588	168965	hypothetical protein	
					168937	169656	hypothetical protein	
					169933	170880	hypothetical protein	
					170952	171146	AlpA family phage regulatory protein	
					171457	171915	hypothetical protein	
					171927	172349	hypothetical protein	
					172666	172935	hypothetical protein	
					173203	173601	single-stranded DNA-binding protein	
					173666	173932	hypothetical protein	
					174279	174500	hypothetical protein	
					175211	176203	integrase	

	176200	177369	integrase
	177371	177796	GNAT family acetyltransferase
	177789	180353	restriction endonuclease subunit M
	180354	180539	hypothetical protein
	180569	181423	transposase
	181474	181740	transposase
	182014	184326	hypothetical protein
	184436	184768	hypothetical protein
	184827	185093	transposase
	185144	185998	transposase
	186641	186970	hypothetical protein
	186960	189299	conjugal transfer protein TraD
	189346	192258	TrwC protein
	192900	193385	hypothetical protein
	193364	194341	hypothetical protein
	194338	196278	hypothetical protein
	196275	197375	hypothetical protein
	197368	199287	hypothetical protein
	199293	200474	hypothetical protein
	200714	201349	peptidase S24
	201964	202284	nuclease
	202626	202835	hypothetical protein
	202837	205539	conjugal transfer protein TraG
	205547	206983	conjugal transfer protein TraH
	206973	207785	conjugal transfer protein TraF
	207782	209506	conjugal transfer protein TraN

	209503	210258	hypothetical protein
	210255	211268	conjugal transfer protein TraU
	211265	211897	type-F conjugative transfer system protein TraW
	211897	212403	conjugal transfer protein TraF
	212400	212780	hypothetical protein
	212773	213189	hypothetical protein
	213189	215738	type-IV secretion system protein TraC
	215735	216349	hypothetical protein
	216346	217215	thiol:disulfide interchange protein DsbC
	217212	218519	conjugal transfer protein TraB
	218519	219274	hypothetical protein
	219271	219840	conjugal transfer protein TraE
	219853	220140	conjugal transfer protein TraL
	220167	220493	hypothetical protein
	220855	221346	hypothetical protein
	221339	221560	transcriptional regulator
	221870	222772	hypothetical protein
	222781	223182	hypothetical protein
	223206	224207	hypothetical protein
	224349	226664	TonB-dependent receptor
	226664	228169	alkaline phosphatase D
	228702	229841	CoA transferase
	230138	231682	AMP-dependent synthetase
	231679	232506	enoyl-CoA hydratase
	232503	233741	thiolase
	233754	234899	acyl-CoA dehydrogenase

					234916	235830	short-chain dehydrogenase
					235992	238175	ligand-gated channel
					238339	239256	LysR family transcriptional regulator
					239376	240356	integrase
					240353	240727	transposase
GI-3	SIGI-HMM	546686	552540	5854	546686	548029	MATE family efflux transporter
					548145	549665	apocarotenoid-15,15'-oxygenase
					549748	550146	very short patch repair endonuclease
					550198	551508	DNA (cytosine-5-)-methyltransferase
					551635	552540	hypothetical protein
GI-4	IslandPath-DIMOB	703710	716209	12499	703710	704000	hypothetical protein
					704058	704321	hypothetical protein
					704348	704725	phage-shock protein
					704722	705018	phage shock protein B
					705025	705870	phage shock protein PspA
					706282	707322	sigma-54-dependent Fis family transcriptional regulator
					707319	707648	hypothetical protein
					707645	707902	hypothetical protein
					708023	708634	hypothetical protein
					708778	709344	ribosome maturation factor
					709344	711017	transcription termination protein NusA
					710998	711738	nucleic acid-binding protein
					711757	714255	translation initiation factor IF-2
					714844	715260	hypothetical protein
					715260	715808	hypothetical protein

					715808	716209	ribosome-binding factor A
GI-5	Islander	825464	960333	134869	825653	826906	integrase
					827114	827710	lytic transglycosylase
					828059	828763	virulence-associated protein E
					828839	833085	methylase
					833190	835163	chromosome partitioning protein ParB
					835458	835670	hypothetical protein
					836054	837730	hypothetical protein
					837727	838905	hypothetical protein
					838983	839720	hypothetical protein
					840014	840943	hypothetical protein
					840955	841395	hypothetical protein
					841615	842550	hypothetical protein
					842620	842883	hypothetical protein
					843158	843556	single-stranded DNA-binding protein
					843622	843885	hypothetical protein
					844228	844449	hypothetical protein
					845044	846216	integrase
					846213	847409	site-specific recombinase phage integrase
					848005	848859	transposase
					848910	849176	transposase
					849190	849633	hypothetical protein
					849614	850078	GntR family transcriptional regulator
					850066	850965	transcription regulator protein
					851129	853648	ATP-dependent DNA ligase

853650	854486	Ku protein
854634	855767	2-alkenal reductase
855819	856319	heat-shock protein
856423	856923	heat-shock protein
857151	857342	hypothetical protein
857499	859118	molecular chaperone GroEL
859176	859490	molecular chaperone GroES
859753	859986	hypothetical protein
859983	860252	protein usg
861052	862113	chemotaxis methyltransferase protein
862322	863419	histidine kinase
863700	864332	integrase
864388	864600	hypothetical protein
864882	865280	single-stranded DNA-binding protein
865358	865576	hypothetical protein
865589	865810	hypothetical protein
866480	866809	hypothetical protein
866799	869138	conjugal transfer protein TraD
869187	872099	TrwC protein
872193	873407	kinase
873404	874027	transcriptional regulator
874600	875475	fructokinase
875475	876617	levansucrase
876765	879236	TonB-dependent receptor
879408	880742	sugar transporter
880753	881790	LacI family transcriptional regulator

	882032	883741	hypothetical protein
	884359	884994	peptidase S24
	885298	885489	hypothetical protein
	885620	885940	nuclease
	886283	886492	hypothetical protein
	886494	889196	conjugal transfer protein TraG
	889206	890642	conjugal transfer protein TraH
	890632	891444	conjugal transfer protein TraF
	891441	893162	conjugal transfer protein TraN
	893159	893914	hypothetical protein
	893911	894924	conjugal transfer protein TraU
	894921	895553	type-F conjugative transfer system protein TraW
	895553	896059	conjugal transfer protein TraF
	896056	896436	hypothetical protein
	896429	896833	hypothetical protein
	896833	899382	type-IV secretion system protein TraC
	899379	899915	hypothetical protein
	899960	900838	thiol:disulfide interchange protein DsbC
	900835	902118	conjugal transfer protein TraB
	902118	902873	hypothetical protein
	902870	903439	conjugal transfer protein TraE
	903452	903739	conjugal transfer protein TraL
	903766	904092	hypothetical protein
	904456	904917	hypothetical protein
	904919	905185	transcriptional regulator
	905233	905619	MerR family transcriptional regulator

905702	906064	hypothetical protein
906073	906717	hypothetical protein
906816	907472	peroxidase
907667	909901	membrane protein
910103	911056	cation diffusion facilitator transporter
911056	911922	hypothetical protein
911919	912548	RND transporter
912545	915040	ATPase
915157	915432	hypothetical protein
915596	918907	cation transporter
918897	920045	metal transporter
920057	921310	metal transporter
921504	921809	hypothetical protein
921879	923807	iron permease
923807	924448	cation transporter
924587	924982	MerR family transcriptional regulator
925009	925500	signal peptidase II
926001	927641	acetolactate synthase
927638	929044	aldehyde dehydrogenase
930014	930424	hypothetical protein
930548	931783	heavy metal RND transporter
931780	933288	RND transporter
933285	936452	cation transporter
936458	936814	hypothetical protein
936871	938415	oxidase
938428	940758	copper-transporting ATPase

					940805	941608	membrane protein
					941640	941969	transcriptional regulator
					942069	942521	phosphatase
					942566	943474	MBL fold metallo-hydrolase
					944040	945392	hydrolase
					945464	946168	hypothetical protein
					946262	946750	hypothetical protein
					946747	947274	hypothetical protein
					947271	947684	membrane protein
					947681	948442	membrane protein
					948543	949895	amino acid permease
					950108	950383	hypothetical protein
					950428	950895	metal-binding protein
					950892	951578	methyltransferase
					951728	952024	hypothetical protein
					952028	952462	hypothetical protein
					952469	953035	RNA polymerase
					953084	954895	copper-binding protein
					954892	956049	copper resistance protein CopB
					956141	956626	hypothetical protein
					956680	957381	membrane protein
					957395	957835	hypothetical protein
					957929	958216	hypothetical protein
					958392	958829	hypothetical protein
					959045	959716	hypothetical protein
GI-6	IslandPath-DIMO	1488326	1502506	14180	1488326	1488832	hypothetical protein

	B						
					1488833	1489300	hypothetical protein
					1489387	1490454	beta-lactamase
					1490451	1492661	DNA ligase (NAD(+)) LigA
					1492658	1493419	hypothetical protein
					1493419	1495083	DNA repair protein RecN
					1495187	1495987	transporter
					1496088	1497272	glutamate 5-kinase
					1497499	1498764	gamma-glutamyl-phosphate reductase
					1498802	1499710	hypothetical protein
					1500000	1502114	hypothetical protein
					1502240	1502506	transposase
GI-7	IslandPath-DIMO B	1617825	1633127	15302	1617825	1619069	hypothetical protein
					1619289	1619666	hypothetical protein
					1619955	1620665	oxidoreductase
					1620662	1621339	hypothetical protein
					1621351	1622103	molybdopterin-binding protein
					1622100	1622474	hypothetical protein
					1622475	1622915	hypothetical protein
					1622976	1624235	cyclopropane-fatty-acyl-phospholipid synthase
					1624331	1625545	argininosuccinate synthase
					1626522	1627058	hypothetical protein
					1627110	1627496	CHRD domain-containing protein
					1628092	1629246	hypothetical protein
					1629352	1629639	hypothetical protein

					1629739	1630005	transposase
					1630056	1630910	transposase
					1630882	1632846	ATP-dependent endonuclease
					1632933	1633127	excinuclease ABC subunit A
GI-8	IslandPath-DIMOB	1855575	1862013	6438	1855575	1855841	transposase
					1855994	1856248	hypothetical protein
					1856245	1856463	hypothetical protein
					1856465	1857061	hypothetical protein
					1856976	1859081	thiol:disulfide interchange protein
					1859114	1860349	ubiquinone biosynthesis protein UbiH
					1860351	1861271	hypothetical protein
					1861324	1862013	uroporphyrinogen III methyltransferase
					1862010	1862888	hydroxymethylbilane synthase
GI-9	Islander	2024110	2029438	5328	2024800	2025195	MerR family transcriptional regulator
					2025265	2025612	hypothetical protein
					2025810	2027141	pyridine nucleotide-disulfide oxidoreductase
					2027138	2027530	hypothetical protein
					2027998	2029227	integrase
GI-10	SIGI-HMM	2144802	2151874	7072	2144038	2144805	molybdopterin biosynthesis protein MoeB
					2144802	2145308	hypothetical protein
					2145284	2145985	NAD-dependent deacylase
					2146095	2146823	4-hydroxy-tetrahydrodipicolinate reductase
					2146834	2147484	endonuclease III
					2147481	2147885	hypothetical protein
					2147895	2149133	glutamate:proton symporter

					2149318	2150454	succinyl-diaminopimelate desuccinylase
					2150484	2150951	transcriptional regulator
					2151044	2151874	endonuclease
GI-11	IslandPath-DIMOB	2178842	2208469	29627	2178842	2179843	LysR family transcriptional regulator
					2179924	2181069	CoA transferase
					2182749	2183603	transposase
					2183654	2183920	transposase
					2183989	2184381	permease
					2184394	2185671	transposase
					2186224	2187153	cobalamin-binding protein
					2187155	2187709	hypothetical protein
					2187721	2189652	TonB-dependent receptor
					2190220	2190702	hypothetical protein
					2190750	2191034	hypothetical protein
					2191189	2192094	hypothetical protein
					2192258	2193460	integrase
					2193789	2195183	chromate transporter
					2195207	2196124	hypothetical protein
					2196604	2198124	ubiquinone biosynthesis protein
					2198464	2200260	hypothetical protein
					2200257	2201132	hypothetical protein
					2201231	2202154	NTP-binding protein
					2202151	2203914	integrase
					2204127	2204420	hypothetical protein
					2204499	2205269	hypothetical protein

					2205301	2206026	hypothetical protein
					2206140	2206811	AAA family ATPase
					2208104	2208469	photosystem reaction center subunit H
GI-12	IslandPath-DIMOB	2820745	2826784	6039	2820745	2821290	hypothetical protein
					2821620	2822768	phage capsid protein
					2822936	2823325	peptidase U35
					2823491	2823799	hypothetical protein
					2823796	2824944	portal protein
					2825002	2825310	endonuclease
					2825432	2826784	ATP-binding protein
GI-13	IslandPath-DIMOB	2937628	3035297	97669	2937805	2939028	integrase
					2939304	2939567	hypothetical protein
					2939723	2940217	hypothetical protein
					2940482	2940676	hypothetical protein
					2940667	2940996	nuclease
					2941283	2941873	murein transglycosylase
					2942477	2942920	hypothetical protein
					2942996	2947243	methylase
					2947348	2949321	chromosome partitioning protein ParB
					2951259	2952191	antirestriction protein
					2952203	2952643	hypothetical protein
					2952863	2953798	hypothetical protein
					2953868	2954137	hypothetical protein
					2954217	2954408	hypothetical protein

2954410	2954808	single-stranded DNA-binding protein
2954876	2955142	hypothetical protein
2956443	2956775	hypothetical protein
2956765	2959080	conjugal transfer protein TraD
2959141	2962065	TrwC protein
2962081	2962716	peptidase S24
2963192	2963806	hypothetical protein
2963811	2964410	hypothetical protein
2964416	2967934	hypothetical protein
2967934	2969121	ATPase AAA
2969118	2969618	hypothetical protein
2969618	2973112	hypothetical protein
2973112	2975607	alkaline phosphatase
2975616	2977697	ATP-dependent Lon protease
2977731	2978867	membrane protein
2978971	2980266	toxin HipA
2980775	2980975	hypothetical protein
2980976	2983678	conjugal transfer protein TraG
2983691	2985127	conjugal transfer protein TraH
2985117	2985932	conjugal transfer protein TraF
2985929	2987653	conjugal transfer protein TraN
2987650	2988399	hypothetical protein
2988396	2989409	conjugal transfer protein TraU
2989406	2990041	type-F conjugative transfer system protein TraW
2990038	2990559	conjugal transfer protein TraF
2990556	2990882	hypothetical protein

2990935	2991312	hypothetical protein
2991312	2993855	type-IV secretion system protein TraC
2993852	2994424	hypothetical protein
2994421	2995293	thiol:disulfide interchange protein DsbC
2995290	2996606	conjugal transfer protein TraB
2996606	2997421	hypothetical protein
2997418	2997987	conjugal transfer protein TraE
2998000	2998287	conjugal transfer protein TraL
2998321	2998653	hypothetical protein
2999018	2999509	hypothetical protein
2999502	2999717	transcriptional regulator
3000207	3000485	hypothetical protein
3000908	3001138	hypothetical protein
3001224	3001502	transposase
3001535	3002416	integrase
3002555	3004141	ubiquinone biosynthesis protein
3004138	3004704	transcriptional regulator
3005484	3006371	peroxidase
3006368	3006781	thioesterase
3007429	3010161	TonB-dependent receptor
3010329	3011399	acyl-CoA dehydrogenase
3011418	3012629	acyl-CoA dehydrogenase
3012698	3013024	(2Fe-2S) ferredoxin
3013053	3014315	cytochrome P450
3014517	3015245	GntR family transcriptional regulator
3015296	3016438	2-nitropropane dioxygenase

	3016520	3017212	hypothetical protein	
	3017209	3018381	transporter	
	3018378	3018854	hypothetical protein	
	3018861	3020111	acyl-CoA dehydrogenase	
	3020111	3021202	acyl-CoA dehydrogenase	
	3021266	3022876	acyl-CoA synthetase	
	3022873	3024510	cyclopentanone 1,2-monooxygenase	
	3024507	3025478	hydrolase	
	3025898	3028834	TonB-dependent receptor	
	3029275	3030762	transporter	
	3030800	3032353	EmrB/QacA family drug resistance transporter	Resistance gene(RGI)
	3032357	3033484	multidrug ABC transporter permease	
	3033593	3033772	hypothetical protein	
	3034108	3034953	ABC transporter substrate-binding protein	

Table S5. Insertion sequences predicted in *E. litoralis* HTCC 2594

orf_name	Similarity_AA	Family	orf_L_end	orf_R_end	orf_bp
Gen_e6014270_1_00150	44.93% ISNpu13_aa2	Tn3	175211	176203	993
Gen_e6014270_1_00151	46.12% ISNpu13_aa2	Tn3	176200	177369	1170
Gen_e6014270_1_00153	100% ISEli1_aa2	IS3 ssgr IS407	181423	180569	855
Gen_e6014270_1_00154	100% ISEli1_aa1	IS3 ssgr IS407	181740	181474	267
Gen_e6014270_1_00156	100% ISEli1_aa1	IS3 ssgr IS407	184827	185093	267
Gen_e6014270_1_00157	100% ISEli1_aa2	IS3 ssgr IS407	185144	185998	855
Gen_e6014270_1_00205	92.94% ISPko4_aa3	IS3 ssgr IS150	240356	239376	981
Gen_e6014270_1_00206	91.93% ISSsp2_aa1	IS3 ssgr IS150	240727	240353	375
Gen_e6014270_1_00237	56.53% TnShfr1_aa1	Tn3	271570	270614	957
Gen_e6014270_1_00821	40.98% ISNpu13_aa2	Tn3	845044	846216	1173
Gen_e6014270_1_00822	44.70% ISNpu13_aa2	Tn3	846213	847409	1197
Gen_e6014270_1_00823	100% ISEli1_aa2	IS3 ssgr IS407	848859	848005	855
Gen_e6014270_1_00824	100% ISEli1_aa1	IS3 ssgr IS407	849176	848910	267
Gen_e6014270_1_00885	58% TnShfr1_aa1	Tn3	911056	910103	954
CtgIS_e6014270__00_3	49.20% TnShfr1_aa1	Tn3	912548	912018	531
Gen_e6014270_1_00998	50.46% ISShvi3_aa1	IS91	1025561	1026481	921
Gen_e6014270_1_01205	48.41% ISShvi3_aa1	IS91	1241534	1240740	795
Gen_e6014270_1_01456	100% ISEli1_aa1	IS3 ssgr IS407	1502240	1502506	267
Gen_e6014270_1_01457	100% ISEli1_aa2	IS3 ssgr IS407	1502557	1503411	855
Gen_e6014270_1_01584	100% ISEli1_aa1	IS3 ssgr IS407	1629739	1630005	267
Gen_e6014270_1_01585	100% ISEli1_aa2	IS3 ssgr IS407	1630056	1630910	855

Gen_e6014270_1_01828	100% ISEli1_aa2	IS3 ssgr IS407	1855524	1854670	855
Gen_e6014270_1_01829	100% ISEli1_aa1	IS3 ssgr IS407	1855841	1855575	267
Gen_e6014270_1_02140	95.09% ISPko4_aa2	IS3 ssgr IS150	2160434	2159454	981
Gen_e6014270_1_02141	93.54% ISSsp2_aa1	IS3 ssgr IS150	2160805	2160431	375
Gen_e6014270_1_02160	100% ISEli1_aa2	IS3 ssgr IS407	2183603	2182749	855
Gen_e6014270_1_02161	100% ISEli1_aa1	IS3 ssgr IS407	2183920	2183654	267
CtgIS_e6014270__00_5	100% ISPst2_aa1	ISL3	2184394	2184483	90
Gen_e6014270_1_02163	100% ISPpu12_aa4	ISL3	2184508	2185671	1164
Gen_e6014270_1_02170	41.06% ISPa43_aa1	Tn3	2192258	2193460	1203
Gen_e6014270_1_02177	45.69% ISChy6_aa2	IS481	2203914	2202151	1764
Gen_e6014270_1_02206	43.93% TnAs1_aa2	Tn3	2226183	2226782	600
Gen_e6014270_1_02481	75.38% ISH7A_aa2	ISNCY	2505088	2505288	201
CtgIS_e6014270__00_4	45.09% TnShfr1_aa1	Tn3	2708567	2709166	600
Gen_e6014270_1_02976	76.08% ISSpma1_aa1	IS3 ssgr IS407	3001224	3001502	279
Gen_e6014270_1_02977	82.49% ISSpwi1_aa3	IS3 ssgr IS407	3001547	3002416	870
CtgIS_e6014270__00_2	81.35% ISEli1_aa2	IS3 ssgr IS407	3007117	3006941	177

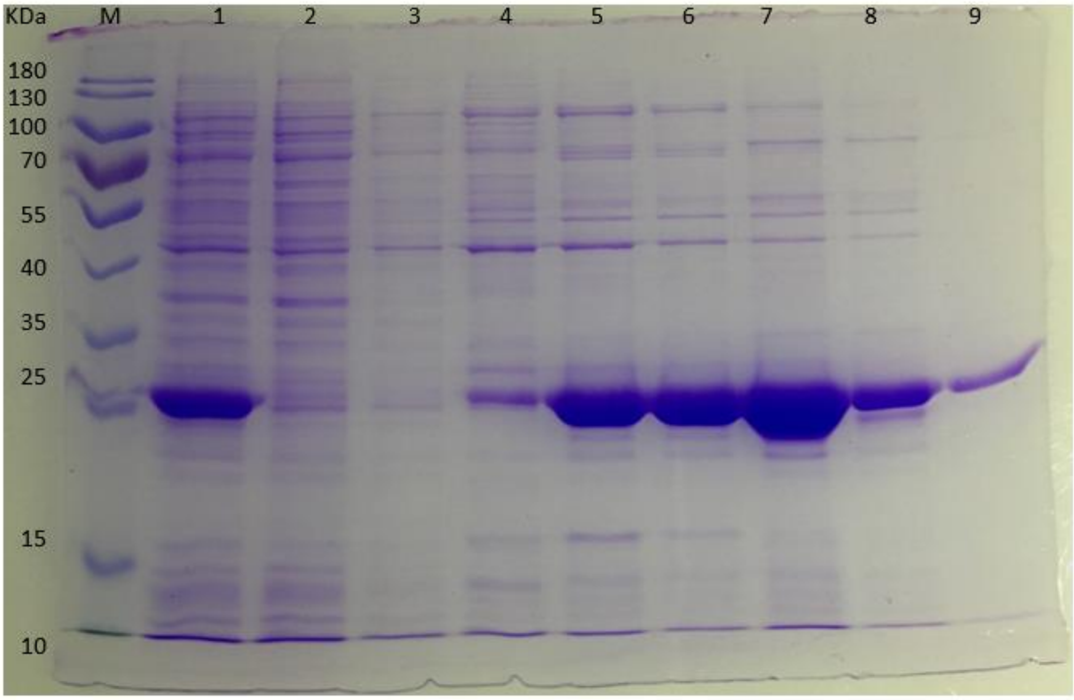

Figure S1. SDS-PAGE of the purified recombinant His-tagged ElBla2. Lane M, Protein Marker (Thermo PageRuler Prestained Protein Ladder, the molecular weight of each band was labelled on the left according to the manufacturer's instruction); Lane 1, Supernatants of the lysed *E. coli* BL21 (DE3) cells which harbors the recombinant plasmid pET28a-*elbla2*; Lanes 2–9, Resultant washed by Tris-HCl (pH 7.4) buffer with 0 mM, 0 mM, 20 mM, 100 mM, 100 mM, 250 mM, 250 mM and 500 mM imidazole, respectively. SDS–PAGE gel concentration was 12 % and was stained by Coomassie R250.

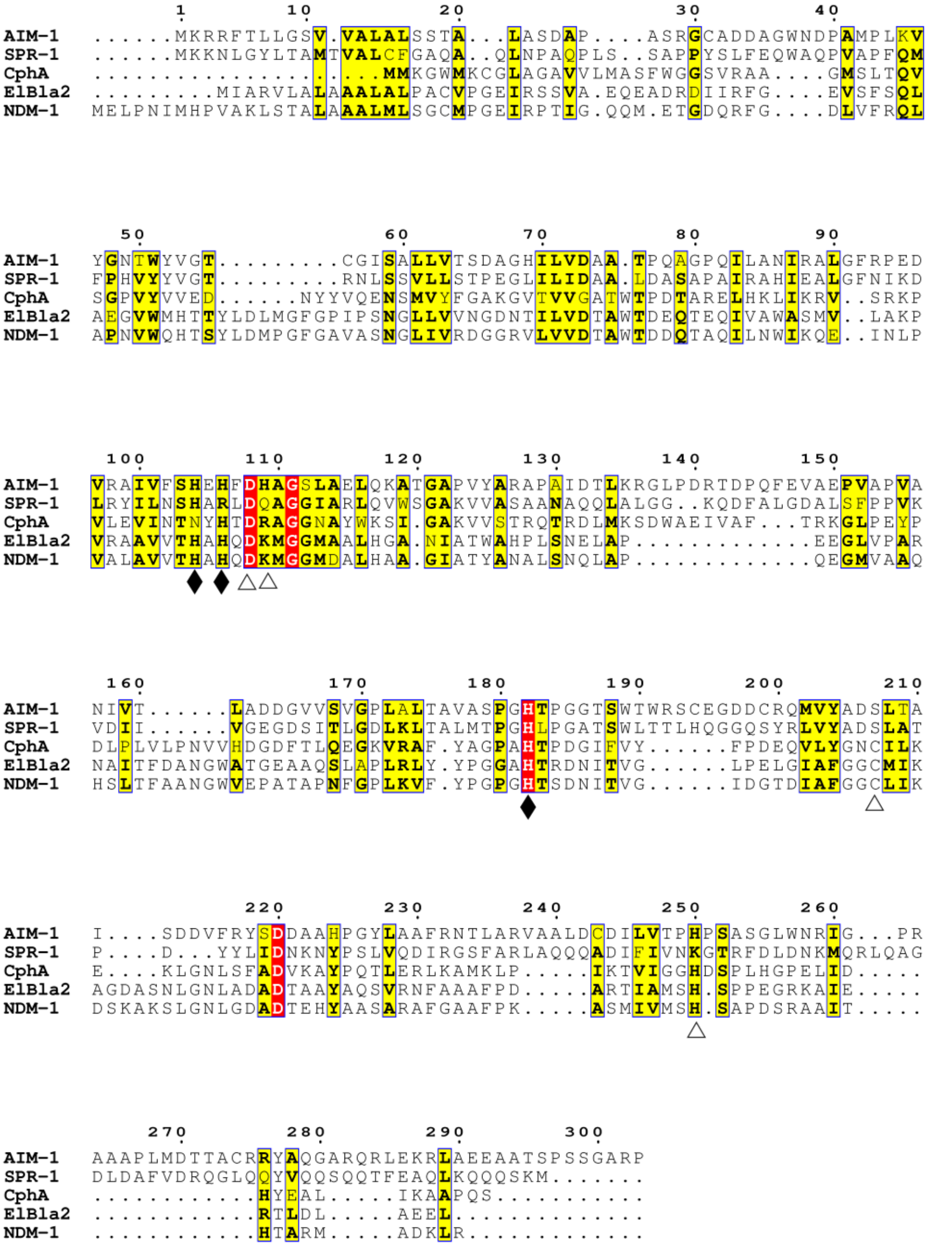

Figure S2. Aligned amino acid sequences of ElBla2 with MBLs from different subgroups. MBLs used in the alignment and their subgroups were NDM-1 (subgroup B1), CphA (subgroup B2), AIM-1 (subgroup B3) and SPR-1 (subgroup B4). Residues involved in the binding of metal ions are indicated in black diamonds (Zn1) and hollow triangles (Zn2). Positions that have a single, fully conserved residue are boxed in red. Conservation between groups of strongly similar properties are boxed in yellow.

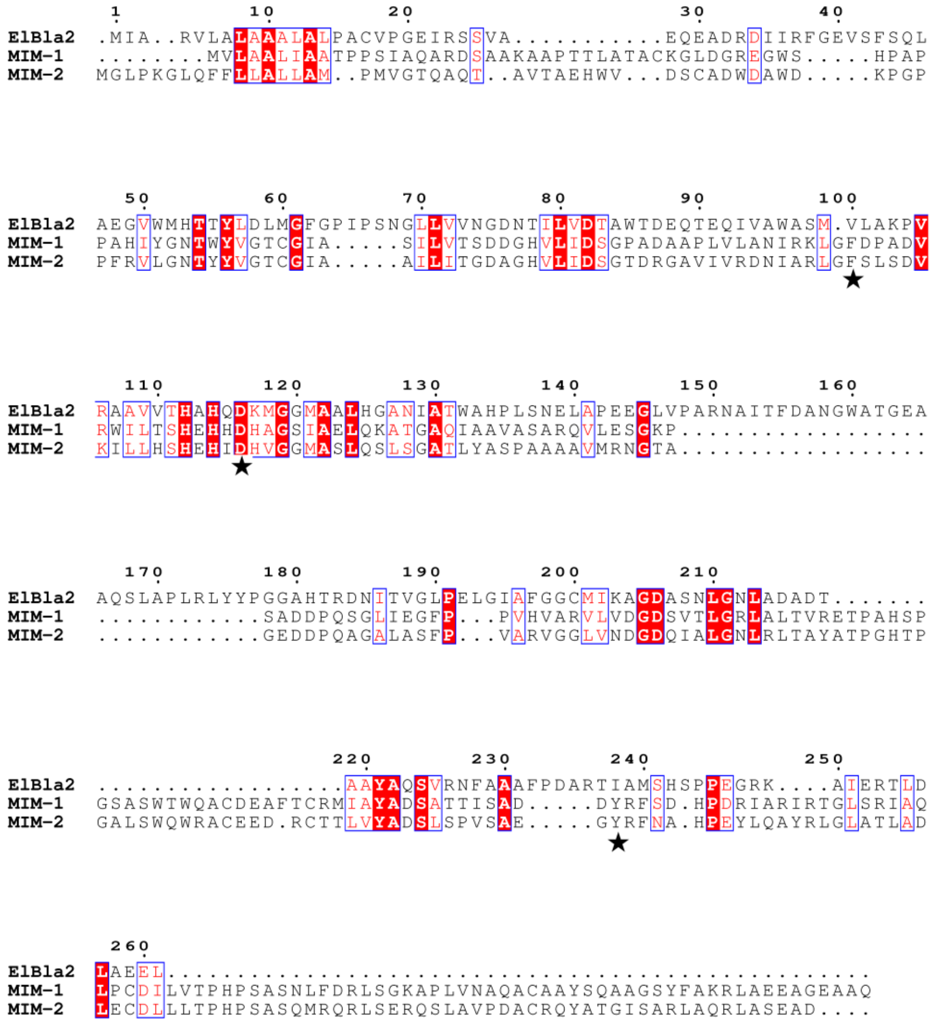

Figure S3. Aligned amino acid sequences of ElBla2 with MIM-1 and MIM-2. Residues known to be critical for lactonase activity are indicated with black stars. Positions that have a single, fully conserved residue are boxed in red.

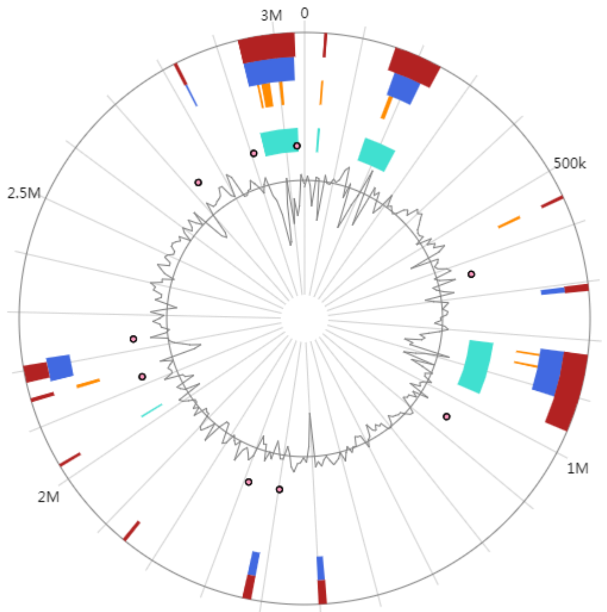

Figure S4. Genomic islands (GIs) predicted in the genome of *E. litoralis* HTCC 2594 by IslandViewer 4. Integrated results are shown in red, GIs predicted via IslandPath-DIMOB, SIGI-HMM and Islander are shown in blue, orange and cyan, respectively.

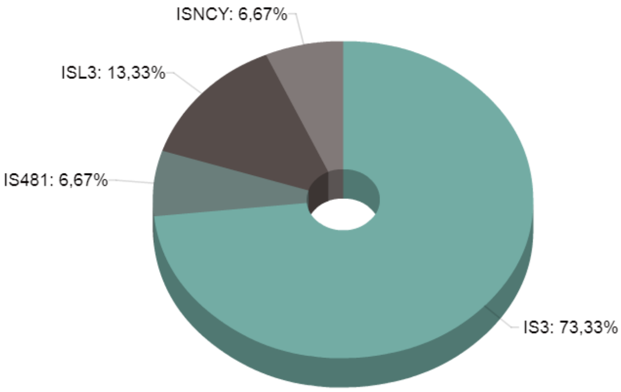

Figure S5. Distribution of insertion sequences (ISs) family in the genome of *E. litoralis* HTCC 2594.

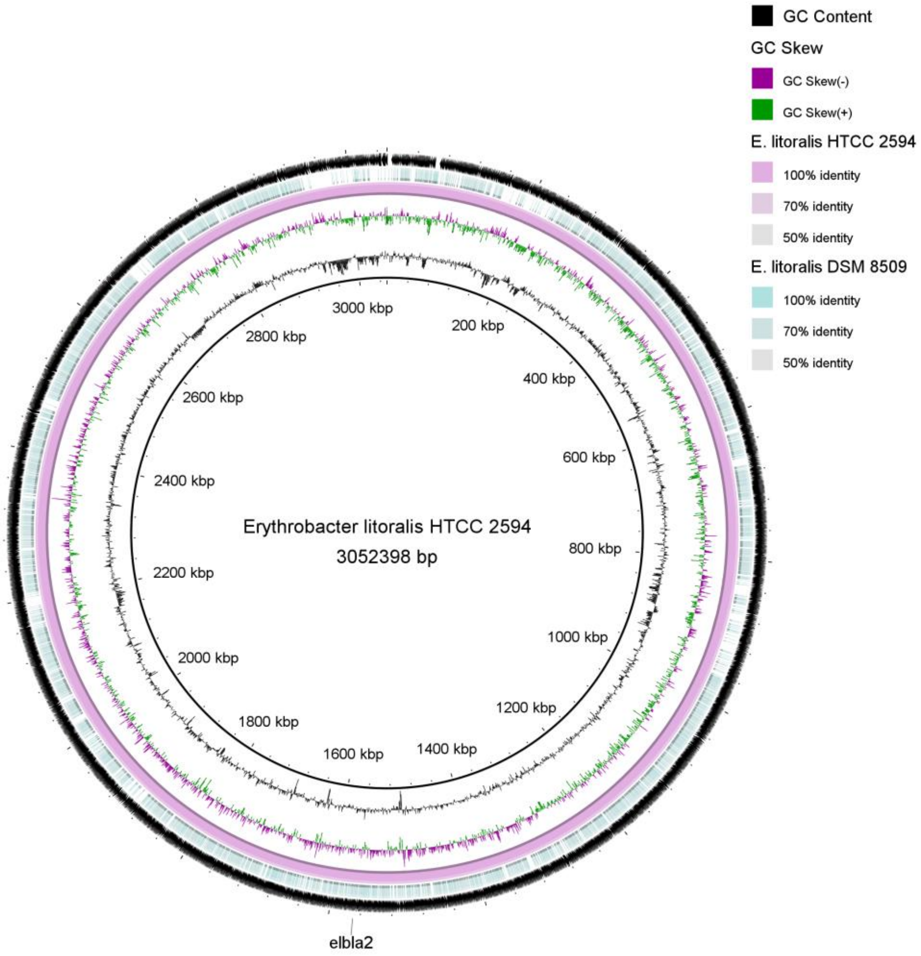

Figure S6. Graphical circular genomic map of *E. litoralis* HTCC 2594 and *E. litoralis* DSM 8509. The pink and blue rings show the BLASTN atlas of the genomes of the two strains. The white and colored regions of the rings indicate regions absent and present, respectively. The CDS encoded for ElBla2 are labeled.
